# *Streptomyces catenulae* as a Novel Marine Actinobacterium Mediated Silver Nanoparticles: Characterization, Biological Activities, and Proposed Mechanism of Antibacterial Action

**DOI:** 10.3389/fmicb.2022.833154

**Published:** 2022-04-28

**Authors:** Maha A. Khalil, Abd El-Raheem R. El-Shanshoury, Maha A. Alghamdi, Jianzhong Sun, Sameh S. Ali

**Affiliations:** ^1^Department of Biology, College of Science, Taif University, Taif, Saudi Arabia; ^2^Botany and Microbiology Department, Faculty of Science, Tanta University, Tanta, Egypt; ^3^Department of Biotechnology, College of Science, Taif University, Taif, Saudi Arabia; ^4^Department of Molecular Medicine, Princess Al-Jawhara Centre for Molecular Medicine, School of Medicine and Medical Sciences, Arabian Gulf University, Manama, Bahrain; ^5^Biofuels Institute, School of the Environment and Safety Engineering, Jiangsu University, Zhenjiang, China

**Keywords:** marine actinobacteria, silver nanoparticles, antibacterial, antioxidant, anti-inflammatory, antitumor

## Abstract

Biosynthesized silver nanoparticles (Bio-SNPs) were synthesized from the marine actinobacterium strain *Streptomyces catenulae* M2 and characterized using a variety of techniques, including UV–vis spectrum, fourier transform infrared spectroscopy (FTIR), energy dispersive x-ray (EDX), transmission electron microscopy (TEM), dynamic light scattering (DLS), surface-enhanced Raman spectroscopy (SERS), and zeta potential. The antibacterial activity of Bio-SNPs alone and in combination with antibiotic was evaluated using a microtiter-dilution resazurin assay against multidrug-resistant (MDR) bacteria. Bio-SNPs’ minimum inhibitory concentration (MIC) against bacterial strains was determined. To assess the synergistic effect of Bio-SNPs in combination with antibiotics, the Fractional Inhibitory Concentration Index (FICI) was calculated. While the safety of Bio-SNPs in biomedical applications is dependent on their use, the *in vitro* cytotoxicity of Bio-SNPs on normal human epithelial colon cells (NCM460) and human colorectal adenocarcinoma cells (CaCo2) were evaluated using the [3-(4,5-dimethylthiazol-2-yl)-2,5-diphenyltetrazolium bromide] (MTT) assay and cell lactate dehydrogenase (LDH) release. The presence of Bio-SNPs was revealed by UV–vis spectroscopy, which revealed a peak in the Surface Plasmon Resonance (SPR) spectrum at 439.5 nm. Bio-SNPs were spherical in shape and small in size (average 33 nm by TEM, 58.8 nm by DLS), with good stability (−30 mV) and the presence of capping agents. Bio-SNPs had MIC values ranging from 2 to 64 μg/ml against the bacteria tested. The MIC for *P. aeruginosa* was the lowest (2 μg/ml). Antibiotics have been shown to have a significant synergistic effect when combined with Bio-SNPs against tested bacteria. Bio-SNPs exhibited dose-dependent cytotoxicity against NCM460 and CaCo2 cancer cells, with the latter exhibiting far greater toxicity than  the  former.  NCM460  and CaCo2  cell   viability   decreased   from  99.3 to 95.7% and 92.3 to 61.8%, respectively, whereas LDH leakage increased from 200 to 215 nmol/ml and 261 to 730 nmol/ml, respectively. The half inhibitory concentrations (IC_50_) for NCM460 and CaCo2 cancer cells were 79.46 and 10.41 μg/ml and 89.4 and 19.3 μg/ml, respectively. Bio-SNPs were found to be biocompatible and to have anti-inflammatory activity. Bio-SNPs are highly appealing for future nanomedicine applications due to their antibacterial and biocompatible properties and their inherent “green” and simple manufacturing.

## Graphical Abstract

Biological applications of silver nanoparticles derived from *Streptomyces catenulae*.

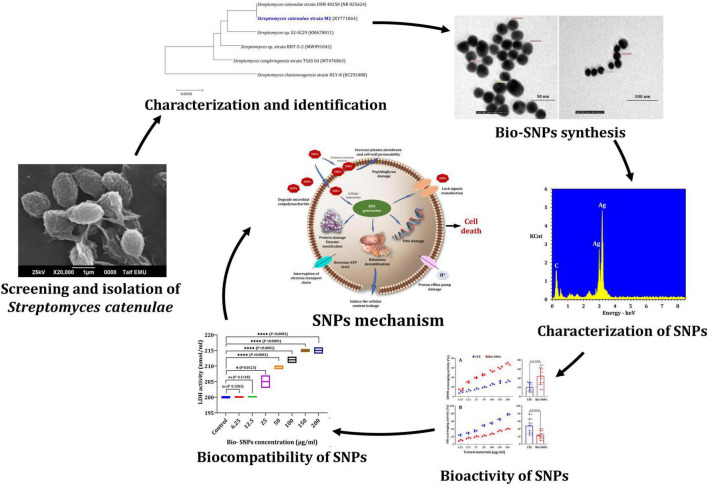

## Introduction

As scientific interest in marine microorganisms has grown in recent decades, actinobacteria and their interesting bioactive components have started to be exploited for their strong biological functions (Sanjivkumar et al., 2019). Marine actinobacteria are a veritable treasure trove of secondary metabolites (Hassan and Shaikh, 2017). Marine actinobacteria are the most valuable prokaryotes competitively and biotechnologically. *Streptomyces*, *Actinomyces*, *Arthrobacter*, *Corynebacterium* and more are representative actinobacteria genera (Manivasagan et al., 2013). Secondary metabolites generated by marine actinobacteria are involved in various biological processes. Marine sediments have recently been found as a unique source of actinobacteria, which produce useful compounds such as antibiotics, enzymes, and nanoparticles (Sanjivkumar et al., 2016, 2018). Actinobacteria have been identified as effective synthesizers of metal nanoparticles in a variety of sources (Balagurunathan et al., 2011). Actinobacteria, including the species *Streptomyces*, can produce a diverse array of bioactive compounds in biomolecules, including antimicrobials. The group has enormous pharmaceutical potential that other microbial groups unmatch. The enormous variety, along with its undervaluation, is a significant factor in enticing researchers to the task of identifying new metabolites (Karthik et al., 2014).

Nanotechnology is a new and fast-developing discipline of research that is concerned with the formation and manufacturing of a variety of nanomaterials using copper, zinc, titanium, magnesium, gold, and silver (Khalil et al., 2017; Shaheen et al., 2019). It is a rapidly growing, interdisciplinary discipline that has a significant impact on the medical, food, agricultural, electrical, and industrial domains, where materials are manufactured at the nanoscale (sizes ranging from 1 to 100 nm) (Elfeky et al., 2020; Nile et al., 2020). Nanoparticles may be synthesized by chemical, physical, or biological processes. In general, physical procedures give low yields, but chemical methods pollute the environment due to the use of precursor chemicals, toxic solvents, and the production of harmful products (Nikzamir et al., 2021; Salem and Fouda, 2021). Consequently, there is a growing demand for environmentally acceptable, safe, efficient, and inexpensive ways of producing nanoparticles that do not emit harmful byproducts during the production procedure (Sharaf et al., 2019; Abdel-Azeem et al., 2020; Ali et al., 2021). Nanoparticles, such as silver nanoparticles (SNPs) maybe more advantageous than bulk counterparts due to their high surface area to volume ratio, allowing for greater interaction with microbes. Bio-SNPs are non-toxic to human cells at low doses and are considered a safe antibacterial agent (Wypij et al., 2018). Furthermore, Bio-SNPs have piqued the interest of entomologists for their potential use in crop protection due to their low toxicity to the environment and humans (Roni et al., 2015; Jafir et al., 2021). Thus, in recent years, the development of nano-scaled particles has become a focus of interest for researchers in the fields of biology, agriculture, and biomedicine (Anwar et al., 2017; Khalil et al., 2021a,b; Zaki et al., 2022), with various studies on the characterization and biomedical properties of silver nanoparticles synthesized by an actinobacterium *Streptomyces olivaceus* (MSU3). An antibacterial agent prevents or eliminates bacterial development. Antibiotic-resistant microbes keep infectious diseases a leading cause of mortality (Ali et al., 2016, 2021; El-Shanshoury et al., 2020a). The number of microbial pathogens that are resistant to antimicrobial is increasing at an alarming pace throughout the globe (Shakoor et al., 2020; Khalil et al., 2021b). As a result, the misuse of antibiotics in the treatment of infectious diseases has aided in the rise of multidrug-resistant (MDR) bacteria (isolates resistant to at least three antimicrobial groups) (Khalil et al., 2015a,b; Sonbol et al., 2015; Ali et al., 2017). Antibiotic effectiveness and resistance to infections have declined, necessitating the emergence of new treatments (El-Zawawy and Ali, 2016; El-Shanshoury et al., 2020b,c; Zaki et al., 2022). A new generation of medications with no side effects is urgently needed to address this issue. Recently, nanoparticle production has been reported in a few actinobacterial strains, including *Nocardiopsis* sp. MBRC-1 (Manivasagan et al., 2013), *Nocardiopsis dasonvillei* (Khalil et al., 2021b), *Streptomyces viridogens* (HM10) (Balagurunathan et al., 2011), *Nocardia farcinica* (Oza et al., 2012).

Cancer is one of the most serious health issues humans confront, with breast cancer being the second largest cause of cancer death in women (Bray et al., 2018). Surgical excision, chemotherapy, and radiotherapy are all conventional treatments for treating cancer disorders, either alone or in combination. However, these therapy modalities are not without considerable adverse consequences. Recently, scientists and researchers have been striving to discover innovative cancer medicines that are non-toxic, affordable, and effective with few side effects (Venugopal et al., 2017; Yesilot and Aydin, 2019). Nanotechnology offers the potential to enhance existing therapeutic techniques while minimizing the toxicity and adverse effects associated with conventional therapies (Pallavi et al., 2022). Certain cytotoxic treatments may disrupt the cell membrane, allowing the contents of the cell to flow out or impair mitochondrial action (Lewinski et al., 2008). It’s interesting to note that various anticancer agents produced by marine actinobacteria have been discovered through their metabolites (Ravikumar et al., 2012). In a dose-dependent manner, *Streptomyces atrovirens*-produced SNPs inhibited MCF-7 breast cancer cells (Subbaiya et al., 2017). Bio-SNPs are now active against a variety of cancerous cell lines, including A549 (Saravanakumar et al., 2019; Pallavi et al., 2022), MCF-7 (Barai et al., 2018), Hella (Wypij et al., 2018), and CaCo2 (Khalil et al., 2021b).

Currently, it looks as if there have been just a few research concentrating on the discovery of bioactive compounds obtained from marine actinobacteria for application as antitumor and antimicrobial agents. To our knowledge, no research has been published on *Streptomyces catenulae* as a new marine actinobacterium strain for synthesizing Bio-SNPs, which have promising antibacterial antioxidant, and antitumor properties. Therefore, a new actinobacterium strain, *Streptomyces catenulae* M2 was isolated from the sea, purified, and genetically identified to be used in the green synthesis of SNPs from silver nitrate. The resulting Bio-SNPs were characterized and tested for antibacterial activity against MDR pathogenic bacteria. 1,1-diphenyl-2-pyridyl-hydrazine (DPPH) was used to test Bio- SNPs’ antioxidant activity and the OH^–^ radical scavenging activity. Finally, the antitumor activity of Bio- SNPs was determined using the CaCo2 cell line (Human colorectal adenocarcinoma) and Human epithelial colon cells (NCM460). In order to assess Bio-SNPs cytotoxicity for potential pharmaceutical and medical applications.

## Materials and Methods

### Bacterial and Cell Culture

Clinical MDR bacterial strains such as *Staphylococcus aureus*, coagulase-negative (CoNs) *Staphylococcus*, *Pseudomonas aeruginosa*, *Escherichia coli*, *Salmonella* sp., *Klebsiella pneumoniae*, and *Proteus mirabilis* were included in the assessment of antimicrobial activity of Bio-SNPs (Khalil et al., 2021b). Human epithelial colon cells (NCM460) were cultured in RPMI-1640 medium. Human colorectal adenocarcinoma cells (CaCo2) were grown in Dulbecco’s modified Eagle’s medium (DMEM). Both cells were cultured in a medium supplemented with 2 mM L-glutamine, 10% fetal bovine serum (FBS), and 1% penicillin-streptomycin. For 24 h, the cultures were incubated at 37°C in a humidified environment containing 5% CO_2_ and 95% air.

### Sample Collection

The heaviest metal tolerant actinobacterium strain denoted as M2 was obtained from the marine water of Jeddah, Saudi Arabia (Khalil et al., 2021b). The isolated strain was subcultured for 7 days at 28°C on starch casein agar (SCA). Following incubation, the isolate was stored at −80°C in a 0.05 M potassium sodium phosphate buffer (pH 7.0) containing 10% glycerol (v/v) as a working strain for future research. The strain was identified using morphological and biochemical features from Bergey’s handbook of determinative bacteriology (Manivasagan et al., 2013).

### Genotypic Characterization

The chosen isolate M2 was characterized molecularly based on 16S rDNA gene sequencing as described by Kumar et al. (2016). The complete genomic DNA of the M2 isolate was extracted according to the manufacturer’s instructions using the TaKaRa extraction kit (Takara, Ōtsu, Japan). The amplification stage was carried out using a pair of universal bacterial primers, 27F and 1492R. The purified amplification products were sequenced at Macrogen Co., in Seoul, South Korea. The sequences were subsequently placed in the Gene Bank.^1^ A BLAST search was used to assess the sequence homology of M2 with the closest related bacterial strains. A phylogenetic tree was constructed using the MEGA 7.0 program.

### Preparation of Cell-Free Extract

An overnight *S. catenulae* M2 culture (1 × 10^5^ CFU/ml, 20 μl) was introduced into 100 ml starch casein broth (SCB) medium and incubated at 28°C/120 rpm for 96 h. Actinobacterium biomass was obtained after 30 min. of centrifugation at 5000 rpm. Two grams of the obtained cells were re-suspended in 30 ml double distilled water (DDW), homogenized, and sonicated for 10 min before incubating in a water bath at 100°C for 10 min. The supernatant was filtered off using Whatman paper No. 1 after coolin it to room temperature.

### Green Synthesis of Silver Nanoparticles

The formation of Bio-SNPs was accomplished by adding 10 ml aqueous CFE to 90 ml AgNO_3_ (1 mM, Sigma Aldrich) solution and incubating for 24 h at 30°C, pH 7.4 and 2000 ± 100 lux. The color changes of a mixture (from colorless to dark brown) were the first indicator of Bio-SNPs synthesis, and these changes in optical properties were quantified using a UV-vis spectrophotometer to scan the spectra between 300 and 800 nm. DDW and organic solvent (ethanol) were used to purify the obtained nanoparticles. Initially, the pH of the reaction mixture (AgNO_3_ and CFE) was changed to a range of 6–9 in order to optimize the reaction conditions that resulted in the maximum yield of Bio-SNPs. Second, AgNO_3_ was mixed with the CFE and kept at a temperature range of 28–40°C for 96 h to optimize the temperature. AgNO_3_ was added to the CFE at concentrations ranging from 1 to 4 mM (final concentration) and incubated at 28°C for up to 96 h to determine the optimal AgNO_3_ concentration (Veerasamy et al., 2011).

### Characterization of Biosynthesized Silver Nanoparticles

#### UV–Visible Spectroscopy

The formation of Bio-SNPs was confirmed by observing a change in the color of the reaction mixture from yellow to dark brown (Siddiqi et al., 2018). UV–vis spectroscopy (Shimadzu No-UV 1800) with a resolution of 1 nm in the 300–800 nm range was used to monitor the Bio-SNPs.

#### Fourier Transform Infrared Spectroscopy

The dried particle of Bio-SNPs was used to produce a pellet by combining it with potassium bromide (KBr) and the existence of IR bands were determined. The scanning data collected by the Fourier transform infrared spectroscopy (FTIR) instrument in the range of 4000–400 cm^–1^ with a resolution of 4 cm^–1^ were used to identify the functional groups present in the sample (Murillo et al., 2018).

#### Energy Dispersive X-Ray

To confirm the existence of silver nanoparticles in the sample, Bio-SNPs were examined using energy dispersive x-ray (EDX) analysis following the procedures reported by Goel et al. (2021).

#### Transmission Electron Microscopy

Transmission electron microscopy (TEM) analysis was used to determine the morphological characteristics of the Bio-SNPs, including their form and size. A drop of synthesized Bio-SNPs solution was loaded onto a copper grid covered with carbon and dried using a vacuum desiccator to prepare the sample for TEM.

#### Dynamic Light Scattering and Zeta Analyses

Dynamic light scattering (DLS) was used to estimate the average size and surface charge of the Bio-SNPs. Prior to analysis, the Bio-SNPs sample (1 mg/ml) was diluted 100 times in Mili-Q water and ultrasonically processed to ensure uniform dispersion of nanoparticles. The substance was then analyzed using a Malvern DLS device (Nano-Zeta Sizer-HT, United Kingdom).

#### Surface-Enhanced Raman Spectroscopy

For surface-enhanced Raman spectroscopy (SERS) measurements, 10 ml of the nanoparticle suspension was sonicated for 20 min after adding 10 ml of DDW. One drop was put onto a 0.2 mm-thick glass substrate using a micropipette and covered for 48 h at room temperature. The optical microscope was equipped with a 100/NA = 0.90 objective lens and was coupled to a Raman spectrometer (WITec alpha 300) (Agressott et al., 2020).

### Biomedical Application

#### Antibacterial and Synergistic Activity of Biosynthesized Silver Nanoparticles

##### Minimum Inhibitory Concentrations

Antibacterial activities of Bio-SNPs were evaluated using resazurin microtiter dilution method using Muller Hinton broth (MHB) media following Clinical and Laboratory Standard Institutes (CLSI, 2018). In the 96-well microtiter plates, assays were performed (in triplicate). The concentration range of Bio-SNPs examined was 0.25–128 μg/ml, and the final concentration of bacteria in each was 1 × 10^8^ CFU/ml. Inoculated 96-well microtiter plates were incubated at 37°C for 18 h. Following incubation, 20 μl of resazurin dye (0.1% w/v in dist. H_2_O) was added to each well, and the plates were maintained at 37°C for 1 h. The change in color from blue/purple to pink suggested that cells were actively metabolizing. Still, the appearance of dark blue indicated that microbes could not grow in microtiter plate wells. Microplate Reader (Bio-Rad Laboratories Inc., Berkeley, CA, United States) detected microbial growth or suppression at 600 nm. On the other hand, the susceptibility of bacteria to TZP was assessed using the resazurin microdilution method (CLSI, 2018). Piperacillin-tazobactam stock concentrations (128/4 μg/ml, tazobactam with fixed concentration at 4 μg/ml) were serially diluted with sterile H_2_O to determine their minimum inhibitory concentration (MIC) alone or combined with Bio-SNPs. Antibiotic concentrations of 0.25/4–128/4 μg/ml were evaluated in triplicate on 96-well microtiter plates. Instead of SNPs suspensions, control studies were conducted using CFE or a 1 mM AgNO_3_ solution in MHB. The MICs were determined as the lowest concentrations of Bio-SNPs to inhibit bacterial growth (the wells remained blue) (Singh et al., 2013).

##### Checkerboard Method

**FIGURE 1:**
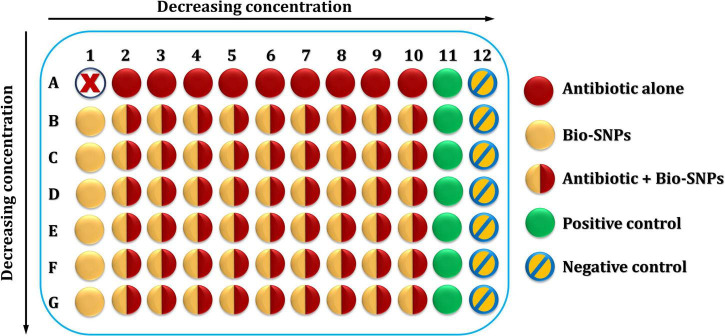
The microtiter plate of the checkerboard method.

Biosynthesized silver nanoparticles with the antibiotic piperacillin-tazobactam (TZP) are studied together using this approach (Fadwa et al., 2021; Zaki et al., 2022). Two-dimensional checkerboard titrations were performed using microdilution broth titrations, with antibiotic and Bio-SNPs concentrations falling in both horizontal and vertical directions (Figure 1). To obtain a starting concentration of 128/4 μg/ml of the antibiotic stock solution, prepare 10 times the stock concentration of piperacillin at 2560 μg/ml, then dilute in a separate test tube containing MHB by twofold increments down to the final concentration (0.5/4 μg/ml) required in this experiment. Add an equivalent amount of tazobactam (80 μg/ml to each diluted tube). Following that, 100 μl of the initial concentration (128/4 μg/ml) was applied to all but one of the wells in column 2 of the microtiter plate. This procedure is then repeated for the remaining dilutions, moving the columns from highest concentration to lowest concentration until column 10. Thereafter, 200 μl of antibiotic dilutions were added to the wells in row A left unfilled, beginning with the 2A well with the initial concentration (128/4 μg/ml) and continuing to column 10, with well 1A remaining empty. Positive and negative controls were utilized in columns 11 and 12, correspondingly. The positive control consisted of 200 μl MHB as well as the bacterial suspension, whereas the negative control, had simply 200 μl MHB.

Biosynthesized silver nanoparticles suspension was accomplished in the following manner: to begin, a rack of seven test tubes was assembled and filled with 1 ml DDW. Secondly, 1 ml of the stock SNPs/DDW suspension with a concentration based on the tested bacterium’s MIC was injected into the first tube and doubly diluted in DDW until it reached the seventh tube. Next, MHB (1 ml) was applied to each test tube to encourage bacterial growth. This resulted in seven tubes with 2 ml of Bio-SNPs suspension in MHB with decreasing concentrations. The concentration was 64 μg/ml or less for the first tube, depending on the organism’s MIC. The wells in columns 2–10 of row B of the same microtiter plate containing antibiotic solutions were then filled with 100 μl of the concentration obtained in the first test tube; the well in column 1 of that row was then filled with 200 μl of the SNPs suspension. The second concentration was filled in the same manner as the first, and so until the seventh concentration. The final microtiter plate included an antibiotic with a decreasing concentration in the horizontal direction and SNPs with a decreasing concentration in the vertical direction, with well 1A remaining empty. Finally, 20 μl of the prepared bacterial suspension was applied to the whole microtiter plate except for column 12th, which served as a negative control.

The checkerboard plates were incubated overnight at 37°C. Then, the antibacterial agents’ MICs were established by comparing test bacteria growth to positive and negative controls. A microplate spectrophotometer at 600 nm was used to measure the microplate’s optical density (OD) before and after incubation. On three different days, each bacterial sample was tested three times. The fractional inhibitory concentration index (FICI) was calculated using Equation (1) to determine the correlation between Bio-SNPs and TZP, where Ab is the antibiotic and Np denotes nanoparticles.


(1) FICI=FIC+AbFICNp

FIC_*Ab*_ = (MIC of Ab in the presence of Np)/(MIC of Ab alone), while FIC_*Np*_ = (MIC of Np in the presence of Ab)/(MIC of Np alone). The FICI value was interpreted following the interpretation ranges. If the result of the FICI is less than or equal to 0.5. If it is greater than 4.0, it is considered synergistic. However, if it is between >0.5 and 1.0, it is considered antagonism, and if it is between >1 and 4.0, it is considered indifference.

##### *In vitro* Antioxidant Activities of Biosynthesized Silver Nanoparticles

The antioxidant properties of Bio-SNPs were determined using the 1,1-diphenyl-2-pyridyl-hydrazine test (Singh et al., 2018) and hydroxyl radical (OH^–^) scavenging activity method (Tanamatayarat, 2016).

##### 1,1-Diphenyl-2-Pyridyl-Hydrazine Scavenging Activity

1,1-diphenyl-2-pyridyl-hydrazine (4 mg; 0.02 mM) was dissolved in 100 ml methanol and kept at 4°C until use. A stock solution (2 ml) was combined with 1 ml methanol containing a range of Bio-SNP concentrations (6.25–200 μg/ml). Methanol and CFE were used as negative controls in this experiment, while ascorbic acid was used as a positive control. After 30 min of incubation in the dark, all mixtures were measured at 517 nm using a UV–visible spectrophotometer.

##### Hydroxyl Radical (OH^–^) Scavenging Activity

The reaction mixture contains EDTA (0.1 ml), FeCl_3_ (0.01 ml), H_2_O_2_ (0.1 ml), deoxyribose (0.36 ml), and Bio-SNPs (1.0 ml). The reaction mixture was then treated with 0.33 ml of 50 mM phosphate buffer (pH 7) and 0.1 ml of ascorbic acid before refrigerating at room temperature for 1 h. In a test tube, 1.0 ml of the incubated mixture was collected. This was followed by adding 1.0 ml TCA (10%) and 1.0 ml TBA (0.5%). The pink color was created, and the pink color’s intensity was measured at 532 nm. The absence of Bio-SNPs in the reaction solution served as a control. The antioxidant scavenging activity was determined using the following formula (2).


(2) $$\text{Scavenging activity}\%=\frac{\mathrm{Control}\,\mathrm{absorbance}-\mathrm{Sample}\,\mathrm{absorbance}}{\mathrm{Control}\,\mathrm{absorbance}}\times 100$$

The amount of Bio-SNPs necessary to scavenge 50% of the radicals (IC_50_) were determined using GraphPad Prism 9.0 (GraphPad Software Inc., La Jolla, CA, United States).

##### Anti-Inflammatory of Biosynthesized Silver Nanoparticles

The anti-inflammatory effect of the bio-SNPs was obtained using the approach reported by Chandra et al. (2012) with slight modifications. Bio-SNPs (2 ml) at varying concentrations (6.25–200 μg/ml) were taken in several test tubes. Acetate buffered saline (2.8 ml; pH 7.0) and 2 ml egg albumin (from fresh hen’s egg) were applied and incubated for 15 min at 28°C. As a control, 100 μg/ml of diclofenac sodium was employed instead of egg albumin. The reaction was generated by incubating the tubes for 10 min. in a water bath set to 70°C. Finally, absorbance at 660 nm was determined using a UV-vis spectrophotometer. The following formula (3) was used to calculate the % inhibition of protein denaturation.


(3) $$\mathrm{Inhibition}\%=\frac{\left(\left(\text{Ac}-\text{AT}\right)\times 100\right)}{\text{Ac}}$$

Where, At = absorbance of test sample; Ac = absorbance of control (diclofenac sodium).

#### Biocompatibility Assay of Silver Nanoparticles

The biocompatibility of Bio-SNPs derived from *Streptomyces catenulae* was determined quantitatively using the [3-(4,5-dimethylthiazol-2-yl)-2,5-diphenyltetrazolium bromide] (MTT) test and lactate dehydrogenase (LDH) leakage assay against NCM460 and CaCo2 cells.

##### [3-(4,5-Dimethylthiazol-2-yl)-2,5-Diphenyltetrazolium Bromide] Test

MTT test was used to determine cell viability after treatment with various Bio-SNPs concentrations (6.25–200 μg/ml) following the method described by Khalil et al. (2021b). NCM460 and CaCo2 cells were seeded at a density of 1 × 10^4^ cells per well onto 96-well plates with suitable culture media and pre-incubated for 24 h at 37°C in a humidified environment containing 5% CO_2_ (Gurunathan et al., 2018). The optical density at 490 nm was determined using a Microplate Reader. The blank wells contained the appropriate growth media with the appropriate concentrations of Bio-SNPs. The cell viability percentage was calculated as previously described (Khalil et al., 2021b). The half inhibitory concentrations (IC_50_) of SNPs were determined using GraphPad Prism 9.0 (GraphPad Software Inc., La Jolla, CA, United States).

##### Lactate Dehydrogenase Activity

Lactate dehydrogenase activity was measured using a colorimetric test kit (K726-500, Biovision, Milpitas, CA, United States), as previously described (Khalil et al., 2021b). After incubating the microtiter plate for 30 min at room temperature in the dark, the absorbance at 490 nm was measured using the Microplate Reader. IC_50_ of SNPs was estimated using GraphPad Prism 9.0 (GraphPad Software Inc., La Jolla, CA, United States).

### Statistical Analysis

GraphPad Prism version (9.0) and Minitab statistical tools were used to evaluate the data (19.2020.1, Minitab Inc., Chicago, IL, United States). Antimicrobial drugs’ antibacterial activity and mortality rate were compared using one-way analysis of variance (ANOVA) with Tukey-Kramer multiple comparisons. An unpaired *t*-test was used to determine the free radical–scavenging activities of the OH^–^ and DPPH radicals. The mean and standard deviation (SD) of three replicates were calculated. Statistical significance is defined as a *P*-value of 0.05.

## Results

**FIGURE 2:**
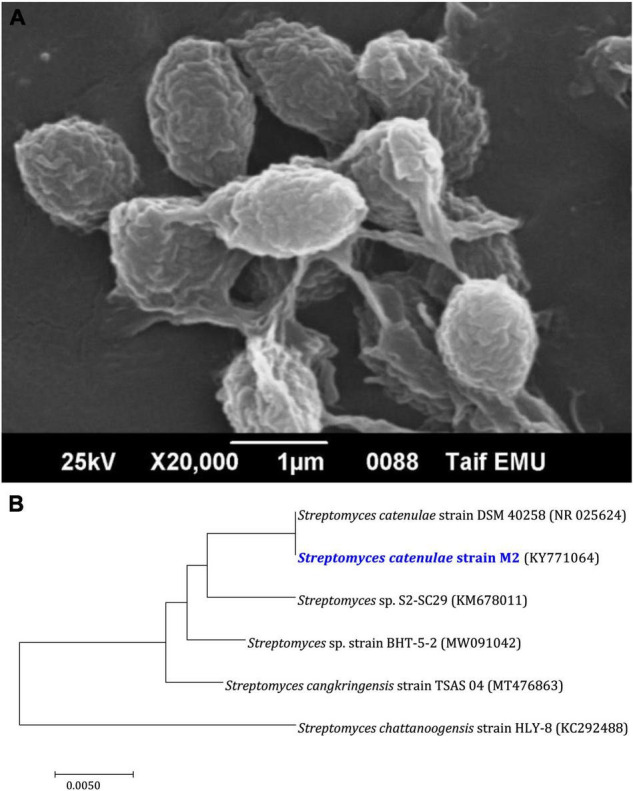
Characteristics of *Streptomyces catenulae* M2. **(A)** Morphological characterization using scanning electron microscope. **(B)** Phylogenetic tree of M2 with its closely related taxa. The bootstrap consensus tree inferred from 1,000 replicates represents the evolutionary history of the taxa analyzed. The scale bar indicates 0.10 substitutions per nucleotide position.

Based on previous research (Khalil et al., 2021b), the most tolerant heavy metal marine actinobacterium M2 was chosen as a new candidate for Bio-SNPs biosynthesis in this study. The sporangia and aerial mycelium of M2 exhibit an irregular structure, as demonstrated by scanning microscope images (Figure 2A). On a molecular level, the M2 isolate was identified as *Streptomyces catenulae* using 16s rDNA PCR. The BLAST analysis and the most closely related phylogenetic relationship indicated a high degree of similarity (98.9%) between *Streptomyces catenulae* strain M2 (KY771064) and *Streptomyces catenulae* DSM 40258 (NR 025624) (Figure 2B).

**FIGURE 3:**
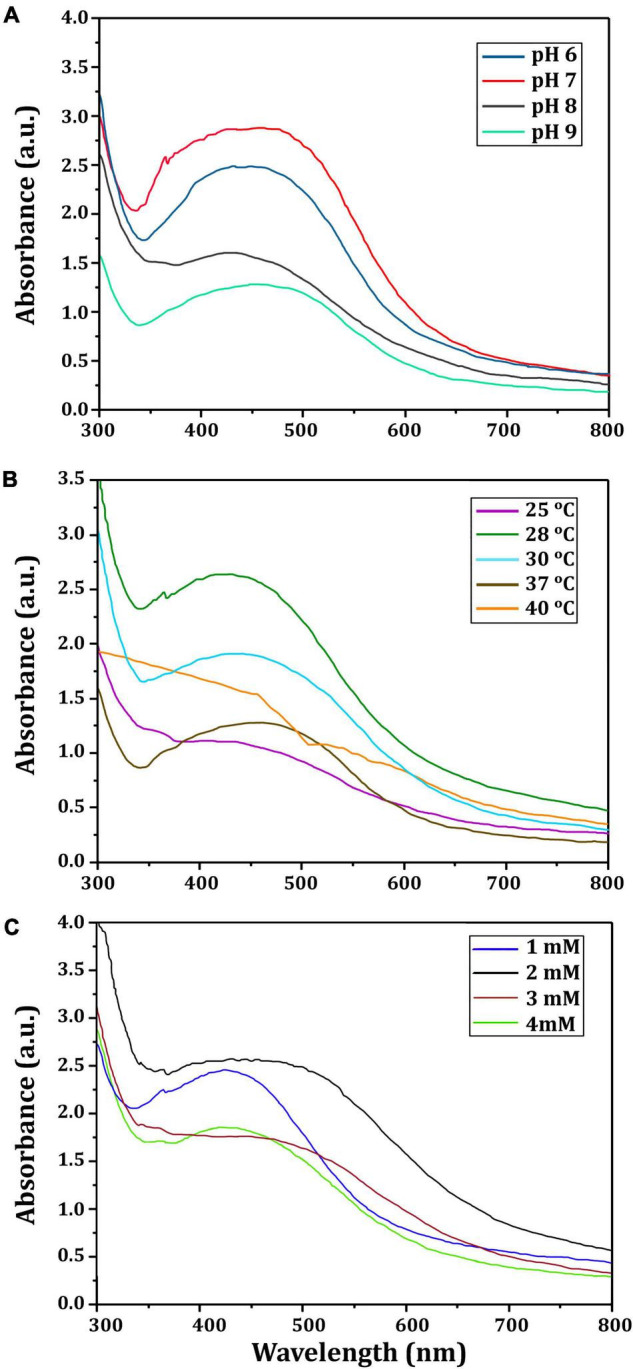
UV-vis spectra of Bio-SNPs obtained at different reaction conditions. **(A)** Different pH value of reaction mixture, **(B)** different reaction temperature (°C), **(C)** different concentration of AgNO_3_ (mM).

**FIGURE 4:**
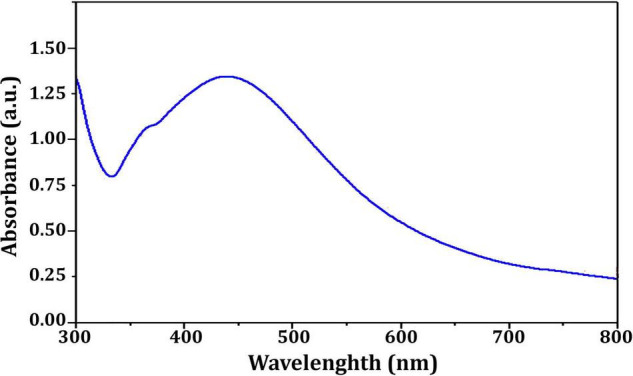
UV-vis spectra of *Streptomyces catenulae* M2-mediated Bio-SNPs.

**FIGURE 5:**
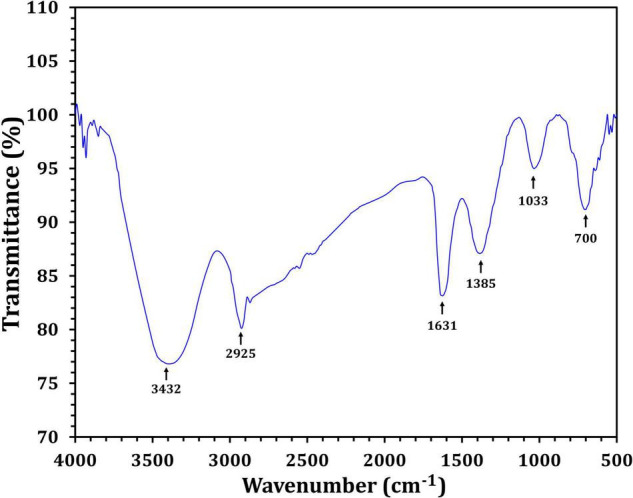
FTIR spectra of *Streptomyces catenulae* M2-mediated Bio-SNPs.

**FIGURE 6:**
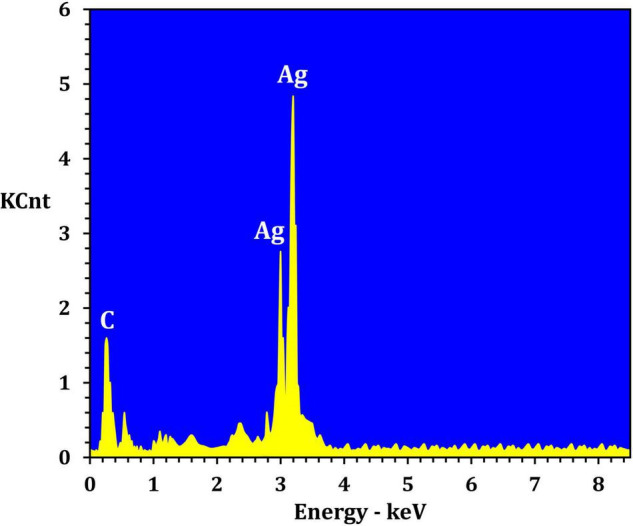
EDX analysis of the *Streptomyces catenulae* M2-derived Bio-SNPs.

**FIGURE 7:**
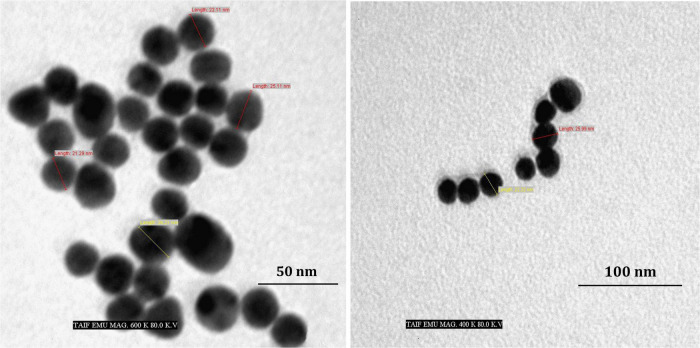
TEM images of Bio-SNPs synthesized by *Streptomyces catenulae* M2.

**FIGURE 8:**
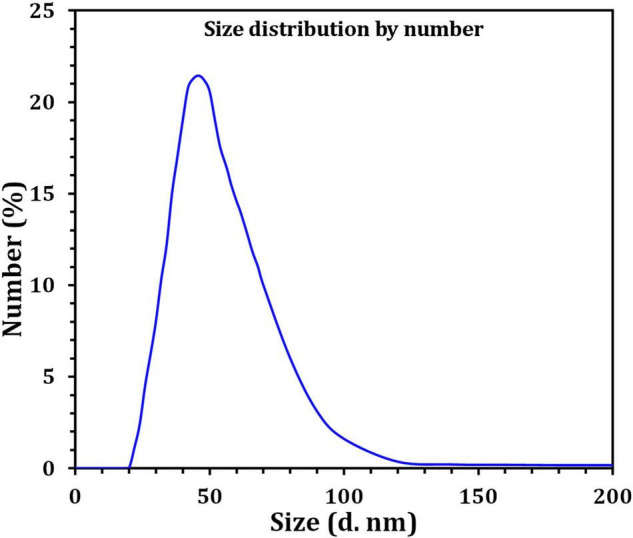
Size distribution by number graph of Bio-SNPs as revealed by DLS.

**FIGURE 9:**
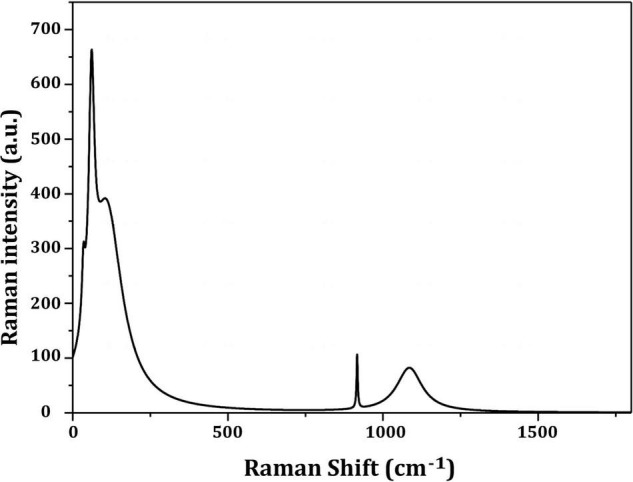
Raman shift of the prepared silver nanoparticles by *Streptomyces catenulae* M2.

Figure 3 depicts the optimization of Bio-SNPs synthesis parameters such as reaction mixture pH, temperature, and AgNO_3_ concentration. An acidic pH (pH = 5) inhibited the synthesis of Bio-SNPs, but the intensity of the color increased as the pH of the solution increased from 6 to 9 (Figure 3A). For Bio-SNPs, a prominent SPR peak, was observed at 460.5 nm with a 2.8929 a.u. absorbance at pH = 7. As shown in Figure 3B, the rate of Bio-SNPs synthesis increased with increasing temperature, as evidenced by a rapid change in the color of the reaction mixture (Figure 3B). The SPR peak at 427.5 nm was visible in the UV-vis spectrum at 28°C, with an absorbance of 2.6469 a.u. However, absorbance did not increase at temperatures above 40°C. The SPR for nanoparticle production varied when the AgNO_3_ concentration was varied at a constant temperature of 28°C (Figure 3C). By adding 1 mM AgNO_3_ to the reaction mixture at a wavelength of 426.5 nm. Above 1 mM AgNO_3_, there was no discernible rise in absorbance. In general, CFE (pH7) and AgNO_3_ solution (1 mM) was incubated at 28°C in the dark on an orbital shaker. The UV-Vis spectrum revealed a single strong SPR signal at 439.5 nm, showing that SNPs were synthesized by CFE (Figure 4). Biomolecules involved in the bioreduction of silver ions (Ag^+^) and the capping of the resultant Bio-SNPs were identified using FTIR analysis. FTIR examination revealed six main functional groups in the wave number range 3432-700 cm^–1^ (Figure 5). Analyzing the CFE’s elemental composition with EDX, silver nanocrystals formed at an optical absorption band peak around 3 KeV, which is typical of metallic SNPs biosynthesized of *S. catenulae*. These findings confirmed the presence of Ag and C groups (Figure 6). The particle size of the produced Bio-SNPs from S. *catenulae* was determined by TEM examination, which revealed that the monodisperse SNPs had a practically spherical shape with a particle size of 33 ± 2.2 nm (Figure 7). Bio-SNPs’ particle size and stability were determined using DLS and zeta potentials. According to the data obtained, Bio-SNPs had a particle size of 58.8 mm and a Zeta potential of −30 mV, as determined by DLS analysis (Figure 8). The presence of silver metal is indicated by the presence of the first complexed peak in the SERS spectrum of Bio-SNPs. In contrast, the presence of the other two complexed peaks indicates the presence of capping materials used in the production and reduction of Bio-SNPs (Figure 9).

Table 1 revealed that the pathogenic bacterial strains tested were TZP resistant, with MIC values ranging from ≥16/4 to 128/4 μg/ml. Bio-SNPs had the lowest MIC value of 2 μg/ml against *P. aeruginosa* and the highest MIC value of 64 μg/ml against *P. mirabilis*. In addition, control wells containing the CFE or AgNO_3_ showed no inhibition. The checkerboard test was used to evaluate the effect of Bio-SNPs and TZP, and the FIC index was calculated by evaluating the degree of interaction between Bio-SNPs and antibiotics against clinical strains (Table 1). The interaction between TZP and Bio-SNPs was synergistic for all Gram negative bacteria tested, with FICI values of 0.253 for *P. aeruginosa*, 0.312 for ESβL *E. coli* and *Salmonella* sp., and 0.5 for *P. mirabilis*, whereas only the *K. pnemoniae* strain showed additive interaction with a FICI value of 0.625. TZP and Bio-SNPs had a synergistic interaction with FICI = 0.5 against *S. aureus*, but an additive interaction with FICI = 1.0 against CoNs *S. aureus* (Table 1).

**TABLE 1:** MIC of TZP, Bio-SNPs, and their combination against MDR bacteria and FICI for the Bio-SNPs/TZP combination.

Bacteria	MIC (μg/ml)			*FICI
	TZP	Bio-SNPs	TZP/Bio-SNPs combination	
*S. aureus*	≥16/4	≤16	4	0.5 (S)
CoNs *Staphylococcus*	≥32/4	≤32	16	1.0 (A)
*P. aeruginosa*	128/4	≤2	≤0.5	0.253 (S)
ESβL *E. coli*	128/4	32	8	0.312 (S)
*Salmonella* sp.	128/4	32	≤8	0.312 (S)
*K. pneumoniae*	64/4	16	≤8	0.625 (A)
*P. mirabilis*	64/4	≤64	≤16	0.5 (S)

*TZP, pipercillin–tazobactam; Bio-SNPs, biosynthesized silver nanoparticles; S, synergy; A, additive.*

*FICI value interpretation: S ≤ 0.5, A >0.5 and ≤1.0.

*The values represent the mean ± SD of three individual observations.*

**FIGURE 10:**
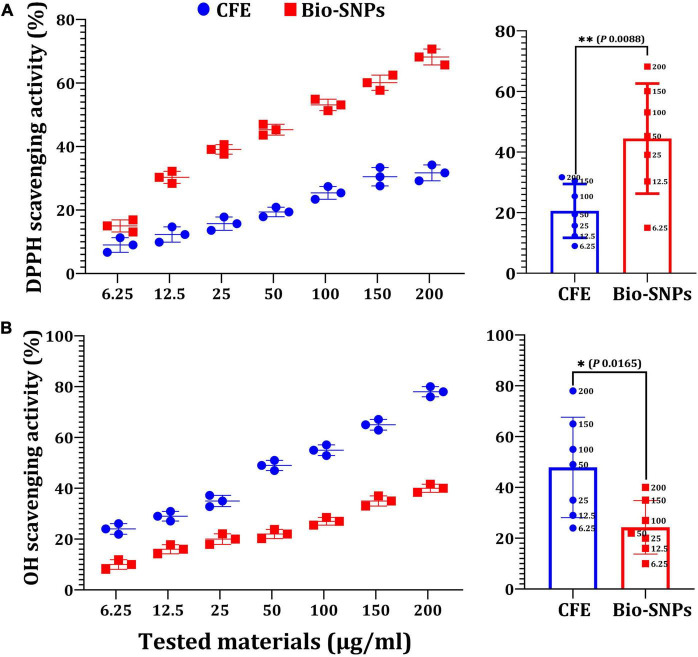
Antioxidant capacity of different concentrations of CFE and Bio-SNPs. **(A)** DPPH radical scavenging activity. **(B)** OH^–^ radical scavenging activity. *P*-values for significantly different mean values, *P < 0.05, **P < 0.01.

*In vitro* antioxidant effects of Bio- SNPs were tested using the DPPH free radical scavenging method. Figure 10A shows that the DPPH scavenging values of Bio-SNPs increased in lockstep with Bio-SNPs’ concentrations. Although the maximum percentage of DPPH activity (68.2%) was found at 200 μg/ml, the lowest percentage of DPPH activity was found at 6.25 μg/ml (15%). Bio-SNPs was a far more effective free radical scavenger than CFE. Bio-SNPs increased antioxidant activity by 2.15 times when used instead of CFE, with a significant difference in *P* = 0.0008 (Figure 10A). CFE and Bio-SNPs had IC_50_ values of 10.99 and 4.59 μg/ml, respectively. The Bio-SNPs demonstrated significant OH^–^ radical scavenging activity, reaching a maximum of 78% at a concentration of 200 μg/ml, compared to 40% for CFE, with a significant difference of *P* = 0.0165 (Figure 10B). The IC_50_ values for CFE and Bio-SNPs were found to be 9.15 and 4.23 μg/ml, respectively. Bio-SNPs have a lower IC_50_ value, indicating their ability to scavenge OH^–^ radicals.

**FIGURE 11:**
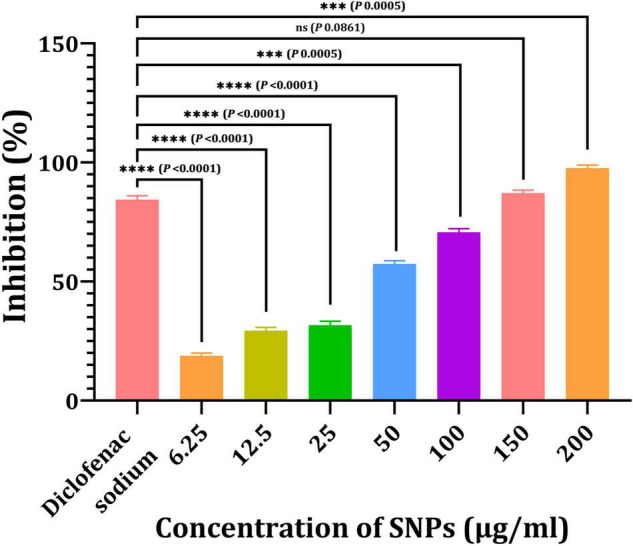
Anti-inflammatory activities of SNPs from *Streptomyces catenulae* M2. *P*-values for significantly different mean values, ***P < 0.001, ****P < 0.0001.

Figure 11 depicts the anti-inflammatory activity of SNPs and standard diclofenac sodium. Bio-SNPs from the candidate strain with concentrations of 6.25, 12.5, 25, 50, 100, 150, and 200 μg/ml exhibited anti-inflammatory activity of 18.76, 29.32, 31.64, 57.32, 70.6, 87.1, and 97.53%, respectively. This revealed that was increasing the concentration of Bio-SNPs has an effect on the percentage of inhibition, whereas standard diclofenac sodium inhibits at an 84.3% rate. The anti-inflammatory efficacy was not significantly different at a Bio-SNPs concentration of 150 μg/ml (*P* = 0.0861). A significant difference in anti-inflammatory activity was observed when standard diclofenac sodium was compared to Bio-SNPs at 6.25 μg/ml (*P* < 0.0001), 12.5 μg/ml (*P* < 0.0001), 25 μg/ml (*P* < 0.0001), 50 μg/ml (*P* < 0.0001), 100 μg/ml (*P* = 0.0005), and 200 μg/ml (*P* = 0.0005) (Figure 11).

**FIGURE 12:**
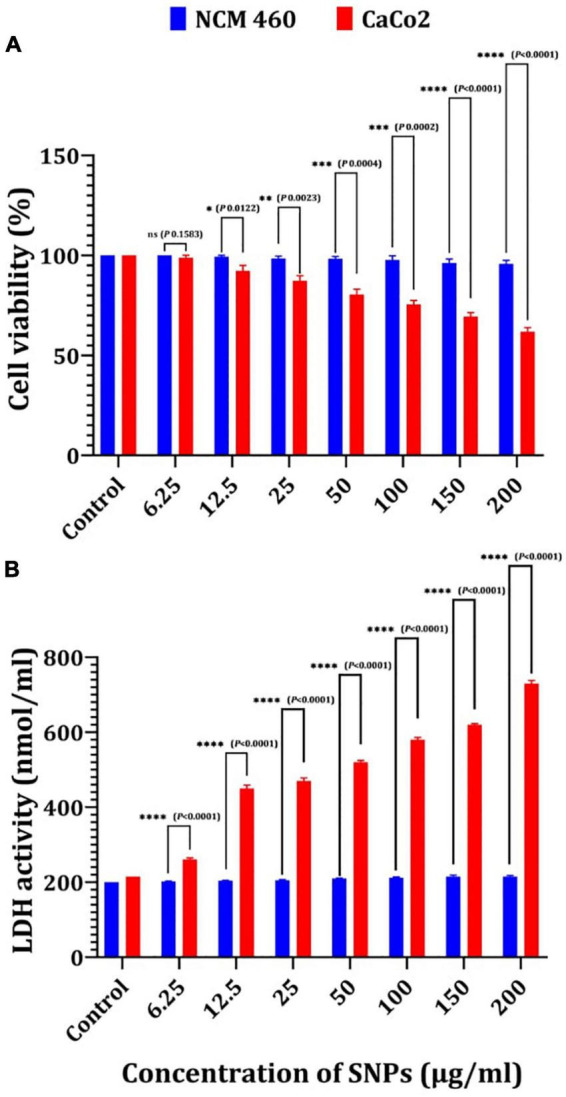
Cytotoxicity of Bio-SNPs toward NCM 460 and CaCo2 cell lines estimated by MTT assay **(A)**, and LDH leakage assay **(B)**. *P*-values for significantly different mean values, *P < 0.05, **P < 0.01, ***P < 0.001, ****P < 0.0001.

NCM460 and CaCo2 cells were used in this study as an *in vitro* model, and the cytotoxicity of Bio-SNPs was determined using MTT and LDH leakage activity. The MTT test was used to determine the cellular activity of two colon cells (Figure 12A). The viability of the NCM460 and CaCo2 cell lines was significantly decreased in the presence of Bio-SNPs at 150 mg/ml and 12.5 μg/ml, respectively. Bio-SNPs doses of 12.5–200 μg/ml exhibited viability of NCM460 cells range of 99.3–95.7%, while decreased CaCo2 cell viability from 92.3 to 61.8% (Figure 12A). The viability percentage of both tested cells was not significantly different at Bio-SNPs doses of 6.25 μg/ml (*P* = 0.1583). When the CaCo2 viability percent as compared to the NCM460 viability percent at different Bio-SNPs concentrations of 12.5 μg/ml (*P* = 0.0122), 25 μg/ml (*P* = 0.0023), and 50 μg/ml (*P* = 0.0004), 100 μg/ml (*P* = 0.0002), 150 μg/ml (*P* < 0.0001), and 200 μg/ml (*P* < 0.0001), a significant difference was found (*P* 0.0122) (Figure 12A). The LDH assay was used to validate the MTT test. The amount of LDH released, a soluble cytoplasmic enzyme, was used to assess the extent of cell membrane breakdown and leakage caused by different nanoparticle concentrations (Figure 12B). There was no significant variation in LDH content in the medium of each Bio-SNPs concentration for NCM460 cells, suggesting that the cell membrane’s integrity was exhibited little damaged. On the contrary, CaCo2 cells stimulated with Bio-SNPs exhibited a dose-dependent increase in LDH leakage compared to control untreated cells (Figure 12B). Following CaCo2 cells treatment with Bio-SNPs dosages ranging from 6.25 to 200 μg/ml, LDH release increased significantly. The LDH activity of NCM460 cancer cells were between 200 and 215 nmol/ml, while that of CaCo2 cancer cells was between 261 and 730 nmol/ml. For growth inhibition against NCM460 and CaCo2 cells, the estimated IC_50_ values were 79.46 and 10.41 μg/ml, respectively. The IC_50_ values obtained from the LDH assay were found to be greater than those obtained from the MTT test (89.4 and 19.3 μg/ml, respectively, for NCM460 and CaCo2 cells). Notably, when all IC_50_ values were compared, it was obvious that the tested Bio-SNPs were more harmful to CaCo2 cancer cells than to normal colon cells NCM460 in terms of metabolic activity and membrane integrity.

## Discussion

The biological production of nanoparticles using actinobacteria opens up a world of possibilities for the development of nanomaterials that might be used as alternative therapeutic agents in a variety of biological applications (Manivasagan et al., 2015; Makvandi et al., 2020). In light of this, a marine bacterium *S. catenulae* M2 isolated from marine water of Jeddah, Saudi Arabia, was used in the present study to produce Bio-SNPs under experimental conditions. Additionally, the synthesized Bio-SNPs were characterized, and they are biomedical properties were evaluated.

A color shift from light yellow to dark brown was noticed, which is the first sign of metal NP production. Additionally, UV–vis spectral examination of the reaction mixtures exhibited peaks around 400 nm, confirming the existence of biosynthesized Bio-SNPs. UV–vis spectroscopy is a powerful and frequently used method for characterizing nanoparticles (Annamalai and Nallamuthu, 2016). The pH, temperature, and AgNO_3_ concentration of the reaction mixture were optimized for the biosynthesis of Bio-SNPs mediated by CFE of *S. catenulae* M2. In terms of pH effect, the results revealed that an acidic pH (pH 5) significantly inhibited Bio-SNPs synthesis, but the color intensity rose as the pH increased from 6 to 9, and Bio-SNPs demonstrated a prominent SPR peak (460.5 nm) at pH 7. It should be emphasized that at extreme pH values, the biomolecules involved in the production of Bio-SNPs might be denatured, and therefore rendered inactive (Singh et al., 2020). Indeed, at acidic pH, all functional groups involved in Bio-SNPs production are positively charged. This is because of the extremely high proportion of protons as well as the knowledge that these functional groups have the less reducing ability at a lower pH. As a result, Bio-SNPs generated under these circumstances are insufficiently stable to avoid agglomeration (Sumi et al., 2017). Larger Bio-SNPs absorb more at longer wavelengths. As a result, as these greater aggregates develop, the range’s absorbance also increases. The SPR peaks seen under neutral and alkaline conditions are compatible with strain M2’s production of Bio-SNPs. Among the temperatures examined in this work, low temperatures provided absorption spectra inappropriate for Bio-SNPs biosynthesis, but high temperatures promoted Bio-SNPs agglomeration. On the other hand, it was discovered that a temperature of 28°C is ideal for producing stable metal NPs. Our findings on the effects of temperature and pH on SNP biosynthesis corroborate those obtained by Balakumaran et al. (2016). The effect of different AgNO_3_ solution concentrations on Bio-SNPs synthesis was investigated. At 426.5 nm, Bio-SNPs were synthesized up to 1 mM AgNO_3_. However, solutions with extremely high concentrations (greater than 2 mM) demonstrated the least amount of silver ion bio-reduction to nanoparticles. This can be explained using enzyme-substrate kinetics; that is, the active site in the key biomolecule responsible for reduction is already saturated with silver ions, and there is no available site for excess ions to be reduced, resulting in no further increase in Bio-SNPs synthesis despite the addition of extra salt (Singh et al., 2013).

Fourier transform infrared spectroscopy studies on Bio-SNPs revealed the presence of specific functional groups in organic molecules that are responsible for the reduction of silver ions to SNPs and the stability of the SNPs (Sanghi and Verma, 2009; Manivasagan et al., 2013). The absorbance peaks at 3432 cm^–1^ are consistent with N–H stretching vibrations (Mohanta and Behera, 2014; Shanmugaiah et al., 2015), whereas the absorbance peaks at 2925 cm^–1^ are consistent with C–H stretching (Karthik et al., 2014). The band at 1631 cm^–1^ is due to the stretching vibration C = C (Annamalai and Nallamuthu, 2016). The peak at 1385 cm^–1^ is attributed to C–H deformation vibration (Deepa et al., 2013), whereas the peak at 1033 cm^–1^ is attributed to C–O stretching vibration (Rai et al., 2015). The peak at 700 cm^–1^ is assigned to aromatic C-H. These findings suggest that Bio-SNPs were capped with organic molecules such as amino acids. EDX analysis was used to validate the chemical compositions of Bio-SNPs. The EDX spectrum of nanoparticles revealed a high peak at 3 KeV for Bio-SNPs, which is typical for metallic SNP absorption (Shahverdi et al., 2007), confirming the presence of SNPs in the CFE.

The TEM analysis was used to characterize the shape and size of silver nanoparticles biosynthesized by *S. rocheii* MHM13. Bio-SNPs were spherical and ranged in size from 22 to 85 nm (Abd-Elnaby et al., 2016). Sanjivkumar et al. (2016) demonstrated that the particle size of the produced SNPs from *S. olivaceus* MSU3 was obtained using TEM examination, describing the morphology of the monodisperse Bio-SNPs as almost spherical with a particle size of 12.3 nm. Herein, the particle size of the produced Bio-SNPs from *S. catenulae* M2 was determined by TEM examination, which revealed that the monodispersed SNPs had a spherical shape with a particle size of 33 nm. Similarly, Sivasankar et al. (2018) reported that the TEM examination of SNPs from *S. violaceus* MM72 indicated a spherical form spanning from 10 to 60 nm. The SNPs from *S. coelicolor* were irregular and with a size range of 20–50 nm as revealed by TEM examination (Manikprabhu and Lingappa, 2013).

Dynamic light scattering was utilized in this experiment to evaluate the particle sizes of Bio-SNPs generated by strain M2. The collected results indicate that the average hydrodynamic diameter is 58.8 nm. The zeta potential analysis provided vital information on the stability of the Bio-SNPs. Bio-SNPs were found to have a negative zeta potential of 30 mV. Numerous studies have shown that metal SNPs with zeta potentials close to 30 mV are extremely stable and have little impact on biological systems (Rezvani Amin et al., 2019). This is due to the strong repulsive contact between the NPs, preventing aggregate formation. Those with a low zeta potential, on the other hand, may cause particle aggregation due to their strong attractive forces (Moore et al., 2015; Bhattacharjee, 2016). The biosynthesized SNPs were shown to be more stable (8.5 mV and 15.7 mV, respectively) than those synthesized by *Streptomyces albidoflavus* and *Streptomyces xinghaiensis* OF1 (Wypij et al., 2018). In general, changes in the size and charge of nanoparticles may be connected to the strain’s specificity, its growth factors, and the parameters utilized during the synthesis method (Donaldson and Stone, 2003). SERS investigates the elevated local fields associated with metallic nanostructures, allowing for detecting very small quantities of material (Feliu et al., 2017).

While the antibacterial modes of action of Bio-SNPs have been widely investigated and debated, they have not yet been completely explained. There are two commonly established antibacterial processes for Bio-SNPs: direct and ion mediated degradation (Qing et al., 2018). The majority of the tested Gram negative bacteria were effectively destroyed by Bio-SNPs, which is consistent with earlier studies (Rathod et al., 2016; Qing et al., 2018; Devagi et al., 2020). This phenomenon has been described by the structural difference between Gram-positive and Gram-negative bacteria’ cell walls, which are mostly constituted of a thick layer of peptidoglycan (Chatterjee et al., 2015; Qing et al., 2018). Several findings, however, contradict this conclusion (Dayma et al., 2019; Bakhtiari-Sardari et al., 2020) or demonstrate varying susceptibility within these bacterial species (Składanowski et al., 2016; Wypij et al., 2018). Nanoparticles combined with β-lactamase inhibitors have recently been an effective and efficient antimicrobial treatment strategy (Naqvi et al., 2013; Panáček et al., 2015; Wypij et al., 2018). This synergism resulted from a binding interaction between antibiotic molecules that contained hydroxyl and amino groups that could easily react with Bio-SNPs. Numerous investigations have shown that the form, size, concentration, and the colloidal state of Bio-SNPs all have a significant effect on their bactericidal activities (Nateghi and Hajimirzababa, 2014). Smaller nanoparticles are more toxic to microbial pathogens than larger sizes, owing to their ease of diffusion, where nanoparticles were most effective when their size was less than 50 nm (Dakal et al., 2016).

**FIGURE 13:**
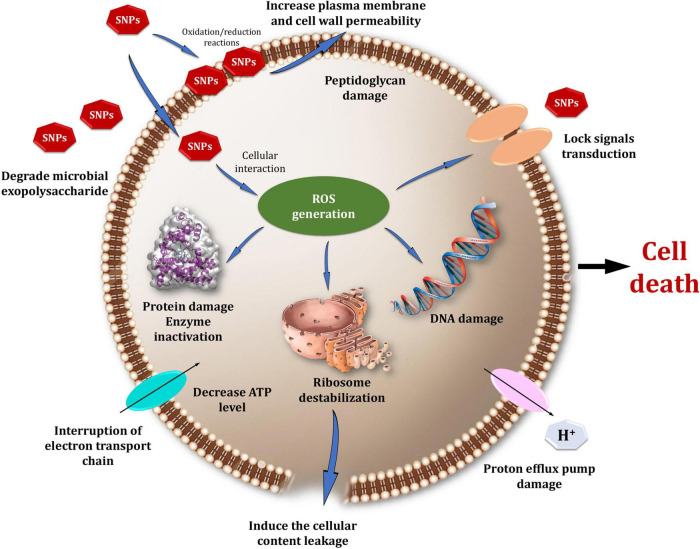
A hypothesized model illustrating possible mechanisms of SNPs’ antibacterial action.

Figure 13 depicts a model that summarizes one proposed mechanism for Bio-SNPs’ antibacterial effect. SNPs exert antimicrobial activity through four distinct mechanisms. First, SNPs bind to the cell wall and membrane’s surface. When Bio-SNPs interact with sulfur-containing proteins in the cell wall, and the structure of the cell wall is irreversibly altered, compromising its strength (Ghosh et al., 2012). As a result, SNPs impair the integrity of the lipid bilayer and the permeability of the cell membrane, which are both necessary for proper control of transport through the plasma (Panáček et al., 2006; Ghosh et al., 2012; Chauhan et al., 2013). Furthermore, SNPs entry into the cell and destruction of intracellular structures and biomolecules (protein, lipids, and DNA) (Morones et al., 2005; Jung et al., 2008; Rai et al., 2012; Chauhan et al., 2013). Toxicity and oxidative stress caused by bio-SNPs are a result of the generation of reactive oxygen species (ROS) and free radicals, which impair cell respiration and growth (Su et al., 2009; Quinteros et al., 2016). Increased ROS levels cause an apoptotic-like response, lipid peroxidation, depletion of antioxidant enzymes, and DNA damage (Lee et al., 2014; Korshed et al., 2016). Additionally, the silver ions generated by nanoparticles add to their biocidal characteristics (Morones et al., 2005; Manivasagan et al., 2013). They may also interfere with microbial cell membrane transport and the release of potassium ions. Increased membrane permeability results in the leakage of cellular contents such as proteins, reduced carbohydrates, and adenosine triphosphate, the cellular energy reservoir (Chauhan et al., 2013; Li et al., 2013).

Antioxidants act as a shield against free radicals, preventing cell damage and playing a critical role in biological applications (Hamasaki et al., 2008). CFE and Bio-SNPs antioxidant activity was determined using a DPPH radical scavenging assay with IC_50_ values of 10.99 and 4.59 μg/ml, respectively. The transformation of violet DPPH to yellow demonstrated Bio-SNPs’ antioxidant activity (Phull et al., 2016; Singh et al., 2018; Ahn et al., 2019). The anti-inflammatory activity test revealed that increasing the concentration of Bio-SNPs (6.25–200 μg/ml) affects the percentage of inhibition, whereas standard diclofenac sodium inhibits 84.3%. According to Sanjivkumar et al. (2016), the anti-inflammatory efficacy of Bio-SNPs from *Streptomyces olivaceus* MSU3 was greatest at 500 μg/ml. Hebeish et al. (2014) demonstrated the anti-inflammatory activity of colloidal SNP solution at a concentration of 250 ppm, with the highest percentage inhibition of 55.9.

The use of Bio-SNPs in biomedical applications, particularly *in vivo*, necessitates an evaluation of their cytotoxic potential (Lewinski et al., 2008; Bin-Jumah et al., 2020; Makvandi et al., 2021). The cytotoxicity of Bio-SNPs was determined in this work utilizing two distinct assays. In the MTT experiment, formazan accumulation directly correlates with mitochondrial activity in living cells, serving as a proxy for cell viability (Manivasagan et al., 2013). On the other hand, when a cytotoxic substance such as Bio-SNPs damages the cell membrane, intracellular LDH molecules are released into the culture media. Thus, LDH leakage is a marker for the decreased cell membrane integrity (Kwan et al., 2016). The IC_50_ values clearly showed that Bio-SNPs were more cytotoxic to CaCo2 cells than NCM460 cells. The IC_50_ of Bio-SNPs tested on the CaCo2 cell line was also determined to be 18 μg/ml (Zein et al., 2020). Additionally, our findings corroborate those of Gurunathan et al. (2018), who discovered that SNPs activate colon cancer cells to release LDH. Thus, our findings appear to be promising, as biosynthesized SNPs are considered an effective antitumor therapy (Firdhouse and Lalitha, 2013; Gajendran et al., 2014; Khalil et al., 2022).

In conclusion, Bio-SNPs’ antibacterial efficacy and stability against MDR pathogenic bacteria were astounding. The presence of a nanoparticle with numerous functional groups enables further modification to increase biological activity, specifically antibacterial, antioxidant, anti-inflammatory, and as well as biocompatibility.

## Data Availability Statement

The datasets presented in this study can be found in online repositories. The names of the repository/repositories and accession number(s) can be found in the article/supplementary material.

## Author Contributions

MK: conceptualization, methodology, formal analysis, data curation, and writing—review and editing. AE-S: validation and visualization. MA: methodology and investigation. JS: investigation, visualization, and validation. SA: formal analysis, data curation, and writing—review and editing. All authors contributed to the article and approved the submitted version.

## Conflict of Interest

The authors declare that the research was conducted in the absence of any commercial or financial relationships that could be construed as a potential conflict of interest.

## Publisher’s Note

All claims expressed in this article are solely those of the authors and do not necessarily represent those of their affiliated organizations, or those of the publisher, the editors and the reviewers. Any product that may be evaluated in this article, or claim that may be made by its manufacturer, is not guaranteed or endorsed by the publisher.

## Footnotes

1
http://www.ncbi.nlm.nih.gov/BLAST/
